# Multi-Tissue Characterization of GILZ Expression in Dendritic Cell Subsets at Steady State and in Inflammatory Contexts

**DOI:** 10.3390/cells10113153

**Published:** 2021-11-13

**Authors:** Molène Docq, Mathias Vétillard, Carmen Gallego, Agnieszka Jaracz-Ros, Françoise Mercier-Nomé, Françoise Bachelerie, Géraldine Schlecht-Louf

**Affiliations:** 1 Inserm U996, Inflammation, Microbiome and Immunosurveillance, Université Paris-Saclay, 92140 Clamart, France; molene.docq@universite-paris-saclay.fr (M.D.); vetillard.mathias@gmail.com (M.V.); carmen.gallego92@gmail.com (C.G.); agnieszka.jaracz-ros@universite-paris-saclay.fr (A.J.-R.); francoise.mercier-nome@universite-paris-saclay.fr (F.M.-N.); francoise.bachelerie@universite-paris-saclay.fr (F.B.); 2 IPSIT SFR-UMS, CNRS, Inserm, Institut Paris Saclay d’Innovation Thérapeutique, Université Paris-Saclay, 92296 Chatenay-Malabry, France

**Keywords:** dendritic cells, *Tsc22d3*/GILZ, skin, inflammation, cancer

## Abstract

Dendritic cells (DCs) are key players in the control of tolerance and immunity. Glucocorticoids (GCs) are known to regulate DC function by promoting their tolerogenic differentiation through the induction of inhibitory ligands, cytokines, and enzymes. The GC-induced effects in DCs were shown to critically depend on increased expression of the Glucocorticoid-Induced Leucine Zipper protein (GILZ). GILZ expression levels were further shown to control antigen-presenting cell function, as well as T-cell priming capacity of DCs. However, the pattern of GILZ expression in DC subsets across tissues remains poorly described, as well as the modulation of its expression levels in different pathological settings. To fill in this knowledge gap, we conducted an exhaustive analysis of GILZ relative expression levels in DC subsets from various tissues using multiparametric flow cytometry. This study was performed at steady state, in the context of acute as well as chronic skin inflammation, and in a model of cancer. Our results show the heterogeneity of GILZ expression among DC subsets as well as the complexity of its modulation, that varies in a cell subset- and context-specific manner. Considering the contribution of GILZ in the control of DC functions and its potential as an immune checkpoint in cancer settings, these results are of high relevance for optimal GILZ targeting in therapeutic strategies.

## 1. Introduction

Appropriate induction of tolerance or immunity is critical for limiting immune-mediated pathologies, like autoimmune diseases and allergies, while favoring protective anti-infectious and -cancer responses. Dendritic cells (DCs) are key players in this equilibrium [1]. They form a heterogeneous family of potent professional antigen presenting cells (APCs) that encompasses ontogenetically- and functionally-defined subsets, currently classified as plasmacytoid (p)DCs, type 1 and 2 conventional DCs (cDC1 and cDC2), and monocyte-derived DCs in both human and mouse [1,2,3]. In the skin, dermal cDCs are further divided in CD103^+^ and CD103^−^ cDC1 cells, CD11b^+^ cDC2 and CD11b^−^CD103^−^ double negative DCs [4]. In addition, Langerhans cells (LCs), a DC-subset ontogenetically related to tissue resident macrophages, survey the epidermis [5]. In tissues, DCs act as sentinels that take up antigens and collect context-dependent signals before migrating to the T-cell zone of lymphoid organs while acquiring antigen presentation capacities. There, depending on their type, the nature of the antigen and the context of its uptake, DCs can either prime and polarize effector T cells, trigger T cell anergy or apoptosis, or favor regulatory T cells (Tregs) expansion and function [6,7,8] in order to orchestrate adequate immune responses. As for skin, dermal cDCs and LCs reach draining lymph nodes (LNs) where they cooperate with LN-resident (res)DC subsets for T cell activation. While migratory (mig)LCs and DCs were shown to induce Th1 (LCs/DC1), Th2 (DC2) or Th17 (all the subsets) responses in many settings, they can also induce tolerance [9,10,11,12]. Hence, identifying the factors that control the tolerogenic versus immunogenic action of DCs and understanding their mechanisms of action is essential for targeted modulation of DCs in therapeutic approaches.

Glucocorticoids (GCs) have long been known as factors that induce immunosuppressive functions in murine and human DCs by driving the expression of inhibitory ligands, cytokines and enzymes and promoting DC capacity to induce and expand Tregs [13,14,15,16,17,18,19]. Tolerogenic DC activation by GCs was shown to critically depend on increased expression of the *Tsc22d3* gene-encoded Glucocorticoid-Induced Leucine Zipper protein (GILZ) [20,21,22,23]. Furthermore, the sole overexpression of GILZ was sufficient to drive the differentiation of tolerogenic DCs favoring Treg proliferation at the expense of effector T-cell priming, both in vitro and in vivo [20,21,24]. Conversely, GILZ deletion in DCs resulted in an increase of their macropinocytic activity while limiting their cross-presentation capacity [25]. Thus, GILZ appears as a molecular switch able to regulate antigen capture by DCs and promote their commitment towards regulatory function [26,27].

GILZ expression levels in DCs have been investigated in some studies. In vitro, in addition to GCs, immunosuppressive cytokines like IL-10 and TGF-β, drugs like mitomycin C and rapamycin or vitamin D3 [17,20,21,28] have been shown to induce GILZ expression in DCs. In vivo, moderate levels of GILZ are constitutively expressed in human blood DCs and murine splenic DC subsets [22,24,25], most likely under the control of endogenous GCs [23]. The injection of hepatocyte growth factor (HGF) was also reported to upregulate GILZ expression in DCs [29], while that of LPS induced rapid GILZ downregulation in splenic DC subsets [25]. Finally, GILZ was reported to be expressed in DCs from the tumor microenvironment (TME) and to mediate the detrimental effects of stress on therapy-induced anticancer responses [23,30]. However, the pattern of GILZ protein expression in DC subsets remains largely unexplored. Thus, the first aim of our present work was to map GILZ expression levels in different DC subsets from both non-lymphoid and lymphoid tissues, at steady state. Our second objective was to characterize whether and how this expression was modulated in different pathologic situations, namely acute and chronic inflammation, as well as in cancer.

Here, by using multiparametric flow cytometry to exhaustively analyze DC subsets in tissue and LNs, we establish that they display heterogenous GILZ levels, the highest expression being detected in LCs and cDC2 in the skin and in LN resDC1 at steady state. Acute inflammation induced by epicutaneous fluorescein isothiocyanate (FITC) application [31] induced GILZ downregulation in skin LCs and cDC2, as well as in migDC1 recovered from skin-draining (SD)LNs. Such GILZ downregulation in skin DCs was however not detected in a context of chronic skin inflammation associated with human papillomavirus (HPV)-induced dysplasia [32,33]. Finally, GILZ was expressed by tumor infiltrating (TI)DCs in a model of HPV-induced tumors, and cancer was associated with opposite modulation of GILZ in cDC1 and cDC2 subsets recovered from tumor-draining (TD)LNs.

Taken together, our results shed light on the differential modulation of GILZ expression levels in DC subsets. Considering the reported contribution of GILZ in the control of T-cell responses and the potential of this protein as an immune checkpoint in cancer settings, these results need to be considered for optimal targeting of GILZ in therapeutic strategies.

## 2. Materials and Methods

### 2.1. Mice

Seven-week-old C57BL/6J and FVB/N mice were purchased from Janvier laboratory (Le Genest, France). The K14-HPV16 (HPV) transgenic mice expressing HPV16 early genes under the control of the keratin 14 promoter were acquired from the NCI mouse repository and maintained on the FVB/N genetic background [32,34]. Mice were bred in the Animex 2 animal facility (IPSIT-SFR, UMS-US31-UMS3679) under specific pathogen–free conditions, in humidity- and temperature-controlled rooms on a 12-h light–dark cycle. The animal experiments were approved by the local Ethics Committee for Animals (C2EA-26; Animal Care and use Committee, Villejuif, France) and follow the French and European guidelines for the use of laboratory animals. Female and male mice were used between 8 and 14 weeks of age.

### 2.2. Epicutaneous FITC Application

One day before the experiment, the belly and flanks of C57BL/6J mice were shaved. A FITC solution (1 mg/mL in acetone; Merck, Molsheim, France) mixed 1:1 with Dibutyl phthalate (Thermo Fischer scientific, Saint-Quentin-Fallavier, France) was prepared. An epicutaneous application of this solution (100 µL/per mouse) was performed on mouse right flanks. Fifteen hours later, mice were euthanized, skin was collected from both the right (+FITC) and the left (-FITC) flanks and processed to obtain cell suspensions for flow cytometry. For each mouse, the left-untreated flank served as an internal control of the right-treated flank.

### 2.3. Tumor Cell Injection

TC-1 tumor cells expressing HPV16 E6 and E7 proteins, derived from primary mouse lung epithelial cells, were obtained from Pr. T.-C. Wu [35]. TC-1 cells were grown in RPMI medium 1640 supplemented with 10% FBS and 100 U/mL Penicillin-Streptomycin (Gibco BRL, Saint-Quentin-Fallavier, France). Two hundred thousand TC-1 cells were injected subcutaneously on the right flanks of mice, in the region proximal to inguinal LNs. Control mice received PBS injection. Mice were monitored daily for tumor growth and sacrificed 17 days after TC-1 cells injection for tumor harvesting and analysis.

### 2.4. Mouse Sample Processing

To obtain single cell suspensions, inguinal or TDLNs were harvested, perfused with a solution of collagenase D (1 mg/mL, Roche, Saint-Quentin-Fallavier, France) and incubated for 30 min at 37 °C. LNs were then mechanically disrupted, and cell suspensions were filtered through 70-μm cell strainers.

For skin cell isolation, tissue pieces were dissected from mouse ear or from depilated (Veet Cream for sensitive skin, used post-mortem) mouse belly and flanks, and processed as previously described [33,36]. Briefly, tissue pieces were incubated for 90 min in dispase (2.5 U/mL, Gibco BRL) at 37 °C. The epidermis and dermis were then separated and cut into small pieces and further incubated for 1 h in a solution of collagenase D (1 mg/mL, Roche) and DNase I (12.5 U/mL, Roche) at 37 °C. Tissue dissociation was then completed using 18-G syringes and samples were further incubated for 15 min at 37 °C before filtration through 70-μm cell strainers.

For tumor cell isolation, tumors were harvested, cut into pieces, and dissociated using gentleMACS™ Dissociator (Miltenyi Biotec, Paris, France) following manufacturer’s instructions.

Cells were counted using LUNA™ automated cell counter (Logo biosystems, Villeneuve d’Ascq, France).

### 2.5. Flow Cytometry

Cells were suspended at 1 to 2.10^7^ cells/mL in PBS-FBS 2% and incubated with anti-CD16/32 monoclonal antibodies (mAbs) for 5 min at 4 °C. Then, cells were further incubated with the appropriate combination of mAbs specific for membrane markers and fixable viability dye (Table 1 and Supplemental Table S1) for 20 min at 4 °C. Intracellular staining (CD207 and GILZ detection) was then performed using the BD Cytofix/Cytoperm™ Fixation/Permeabilization kit, following furnisher’s instructions. The GILZ specific CFMKG15 antibody can potentially recognize all GILZ isoforms, but only GILZ1 has been reported in DCs so far [26].

Data were acquired using a LSR Fortessa flow cytometer (BD Biosciences, San Diego, CA, USA) and further analyzed with FlowJo Software (Tree Star Inc., Ashland, Or, USA). Fluorescence minus one (FMO) controls were used to determine the positivity threshold of CD64, MHC II, CD11b, CD103 and CD207 staining. Isotype control was used to validate GILZ staining. For the determination of GILZ expression levels, delta mean fluorescence intensity (MFI) were calculated by subtracting the MFI obtained with the isotype control in each subset to that obtained with the anti-GILZ mAb. When the experiments pooled were acquired with different LSR Fortessa settings, the delta MFIs obtained in each experiment were normalized to the mean of the delta MFIs of one DC subset as indicated in the figure legends. For all the experiments performed on skin and SDLNs, LCs and migLCs were chosen as reference, respectively, as they correspond to the same DC subset in two localizations and are defined by the positivity for several markers. For the experiments conducted on TDLNs and spleens from tumor-bearing mice, resDC1 were chosen as the reference, as this subset is present in both organs and defined by the positivity for several markers.

### 2.6. Histology

Ear biopsies were fixed in 4% buffered paraformaldehyde (Thermo Fisher Scientific) for 24 h. They were further embedded in paraffin and cut into 5-μm-thick sections. After deparaffination, the sections were rehydrated and stained with hematoxylin-eosin (HE). A NanoZoomer 2.0-RS digital scanner (Hamamatsu, Massy, France) was used to scan the slides with the 40× objective and images were analyzed using NDP.view2 software (Hamamatsu, Massy, France). Measures of epidermis and dermis thickness were performed in at least 10 fields for each sample.

### 2.7. Statistical Analysis

Statistical tests were conducted using Prism software (Version 9.1.2; GraphPad Prism, San Diego, CA, USA). For each set of experiments, the D’Agostino-Pearson test was used to determine whether the data were normally distributed, which was not the case. The non-parametric Mann–Whitney U and Kruskal–Wallis tests were used to compare two or multiple groups, respectively. When Kruskal–Wallis test was used, a correction for multiple comparisons was performed using the Dunn’s test. Significance was defined as: *p* < 0.05 *, *p* < 0.01 **, *p* < 0.001 ***, *p* < 0.0001 ****. The numbers of samples and of independent experiments are indicated in the figure legends, as well as the test used for each comparison.

## 3. Results

### 3.1. LCs Express the Highest Levels of GILZ among Skin DC Subsets

**Figure 1:**
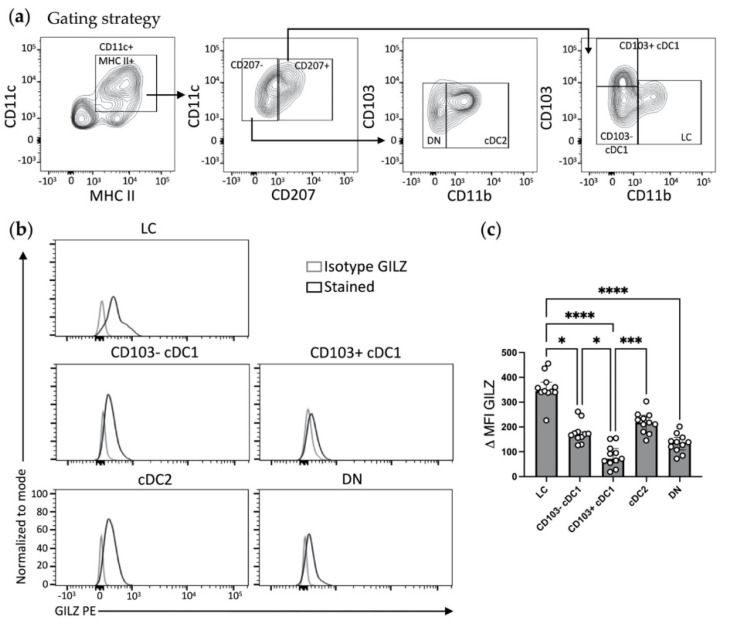
Assessment of GILZ expression levels in skin DC subsets at steady state. (**a**,**b**) Flow cytometry analysis of skin LCs, CD103^−^ cDC1, CD103^+^ cDC1, cDC2 and double negative (DN) DCs. (**a**) Gating strategy for DC subset identification among alive single cells after exclusion of CD64^+^ macrophages and/or CD3^+^ T lymphocytes. (**b**) Representative histograms showing GILZ expression levels in LCs, CD103^−^ cDC1, CD103^+^ cDC1, cDC2 and DN DCs. Isotype controls are shown in gray (isotype GILZ) and GILZ expression in black (Stained). (**c**) Bar graph recapitulating GILZ expression levels, expressed as median with interquartile range of delta MFIs in skin DC subsets. Open symbols represent the values for individual mice. Data are from 2 independent experiments with a total of *n* = 11 mice (7 male and 4 female mice, C57BL/6J background). Statistical analysis was performed using the non-parametric Kruskal–Wallis test to compare all the groups. Significance was defined as: *p* < 0.05 *, *p* < 0.001 ***, *p* < 0.0001 ****.

To characterize GILZ expression in skin DCs at steady state, skin biopsies were taken from the flanks of C57BL/6J mice and cells were isolated by combining enzymatic digestion with mechanical tissue dissociation [33,36]. We used a gating strategy excluding CD64^+^ macrophages and allowing for the identification of five skin DC subsets, including LCs, CD103^−^ cDC1, CD103^+^ cDC1, cDC2, and CD207^−^CD11b^−^ double negative (DN) dermal DCs [4], based on the differential expression of the CD11b, CD103 and CD207 markers (Table 2 and Figure 1a).

Different levels of GILZ were detected in skin DC subsets (Figure 1b,c). LCs expressed the highest levels of GILZ, the detection being twice to thrice fold higher than in CD103^−^ and CD103^+^ cDC1 and DN DCs. In cDC2, GILZ levels tended to be lower than in LCs but higher than in CD103^−^/CD103^+^ cDC1 and DN DCs, albeit differences were not statistically significant except for CD103^+^ cDC1. The latter DC subset expressed minute amounts of GILZ. Altogether, these results show that the levels of GILZ expression is heterogeneous in skin DC subsets at steady state.

### 3.2. ResDC1 Express the Highest Levels of GILZ among LN DC Subsets

**Figure 2:**
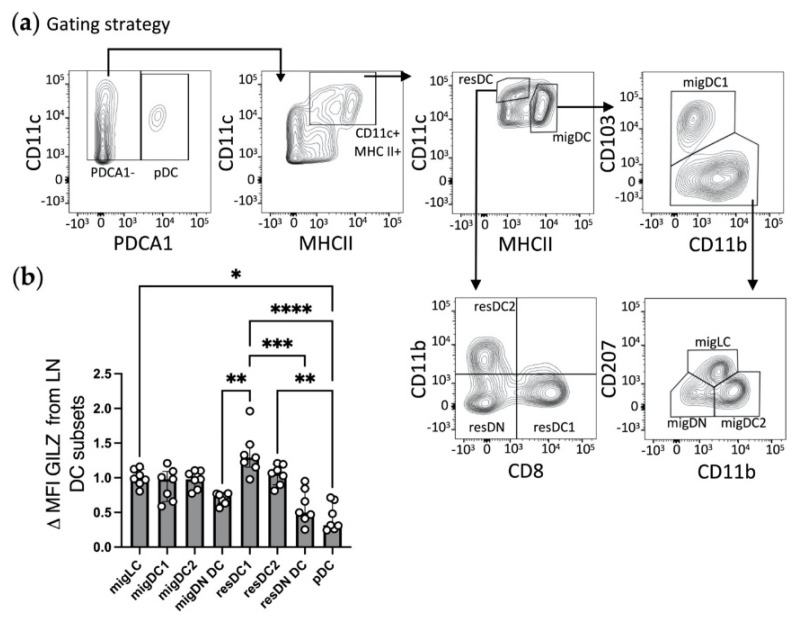
Assessment of GILZ expression levels in LN DC subsets at steady state. (**a**,**b**) Flow cytometry analysis of migratory (mig)DC subsets (migLCs, migDC1, migDC2, and double negative migratory (migDN) DCs), resident (res)DC subsets (resDC1, resDC2 and resident double negative (resDN) DCs) and pDCs. (**a**) Gating strategy for DC subset identification among alive single cells after exclusion of CD3^+^ T lymphocytes. (**b**) Bar graph showing GILZ expression levels in LN DC subsets as median with interquartile range of normalized delta MFIs. Data were normalized relative to migLCs and expressed as folds. Open symbols represent the values for individual mice. Data are from 2 independent experiments with a total of *n* = 7 mice (male mice, C57BL/6J background). Statistical analysis was performed using the non-parametric Kruskal-Wallis test to compare the different groups. Significance was defined as: *p* < 0.05 *, *p* < 0.01 **, *p* < 0.001 ***, *p* < 0.0001 ****.

We next investigated GILZ levels in DC subsets from SDLNs, where skin DCs migrate and join LN-resident (res)DCs as well as pDCs. We used a gating strategy allowing for the identification of 4 migratory DC (migDC) subsets [37], namely migLCs, migDC1, migDC2 and migDN DCs (Table 3 and Figure 2a). In addition, we also analyzed resDC subsets, i.e., resDC1, resDC2 and resDN DCs, as well as pDCs.

As opposed to what was observed in the skin, GILZ expression levels were similar in the migLC/DC subsets recovered from SDLNs (Figure 2b and Supplemental Figure S1). The analysis of LN resDC subsets revealed that resDC1 displayed the highest levels of GILZ expression, while resDN DCs expressed very low GILZ levels, as pDCs did. ResDC2 expressed slightly lower GILZ levels than resDC1, albeit the difference did not reach statistical significance. Finally, the comparison of migDCs/LCs with resDCs revealed that migLCs, migDC1, migDC2 and resDC2 expressed similar GILZ levels. Taken together, these results show that SDLN DC subsets express GILZ at various levels in the steady state, with overall higher levels in resDC1/2 and migDCs/LCs than in resDN DCs and pDCs.

### 3.3. GILZ Expression Levels Are Reduced in a Subset-Dependent Manner upon Acute Skin Inflammation

**Figure 3:**
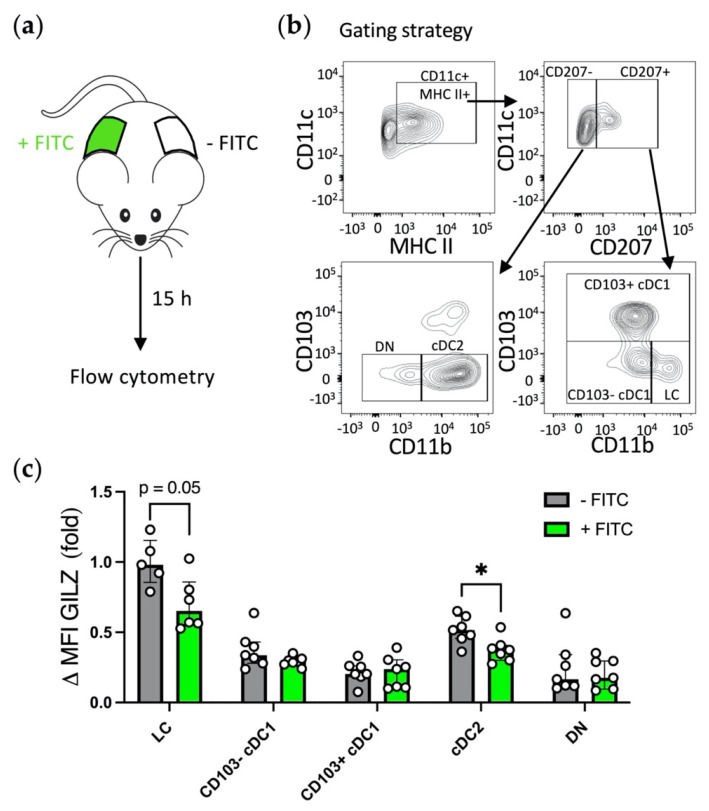
Assessment of GILZ expression levels in skin DC subsets upon acute inflammation. (**a**) A FITC solution was applied on the shaved right flanks of mice. After 15 h, skin from both the right (+FITC) and left (-FITC) flanks was collected and digested to obtain cell suspensions for flow cytometry analysis. For each mouse, the skin from the left-untreated flank served as control for the right-treated flank. (**b**) Gating strategy for skin LC, CD103^−^ cDC1, CD103^+^ cDC1, cDC2 and double negative (DN) DC identification among alive single cells after CD3^+^ T lymphocytes and CD64^+^ macrophages exclusion. (**c**) GILZ expression levels in skin DC subsets. Data were normalized relative to FITC^−^ LCs and are expressed as median with interquartile range of normalized delta MFIs. Open symbols represent the values for individual mice. For LCs, there were not enough cells for MFI analysis for one or two samples, therefore only 5 and 6 symbols are depicted for FITC^−^ and FITC^+^ LCs respectively. The results are from 2 independent experiments with a total of *n* = 7 mice (male mice, C57BL/6J background). Statistical analysis was performed using the nonparametric Mann–Whitney U test to compare GILZ levels in DC subsets from non-treated and FITC-treated flanks. Significance was defined as: *p* < 0.05 *.

We next sought to determine whether GILZ expression level in skin DC subsets is altered in the context of acute inflammation. To investigate this question, we used a FITC painting approach, known to trigger local skin inflammation [31]. A solution of FITC in acetone and dibutyl-phthalate was applied on the shaved right flanks of mice. Fifteen hours later, skin biopsies from both the FITC-exposed (+FITC) and contralateral non-exposed (-FITC) flanks were recovered for flow cytometry analysis of DC subsets (Figure 3a,b). We first assessed whether FITC application was associated with local inflammation by measuring the levels of class II MHC molecule (MHC II) expression, used as maturation marker, in DCs, after exclusion of CD64^+^ macrophages. FITC application induced an increase of MHC II levels in LCs, CD103^+^ cDC1 and CD103^−^ cDC1 as expected, while it was not the case in cDC2 and DN DCs (Supplemental Figure S2a). We next evaluated GILZ expression in skin DC subsets (Figure 3c and Supplemental Figure S2b). GILZ expression levels were reduced in LCs and cDC2 from FITC-exposed skin as compared to their controls but were preserved in the CD103^+^/CD103^−^ cDC1 and DN DC subsets (Figure 3c and Supplemental Figure S2b). Thus, skin acute inflammation induces a diminution of GILZ expression in the skin DC subsets that express the highest levels of GILZ, independently of MHC II expression levels.

### 3.4. Acute Inflammation-Exposed migDC1 Recovered from SDLNs Display Reduced GILZ Expression Levels

**Figure 4:**
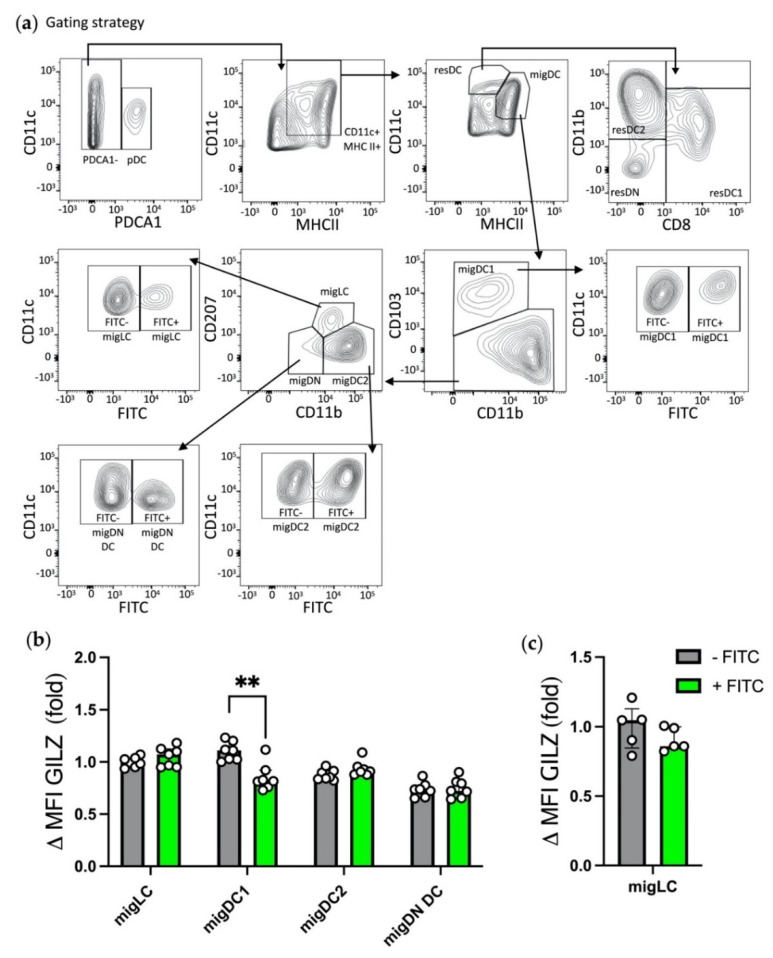
Assessment of GILZ expression levels in LN DC subsets in the context of FITC-induced acute inflammation. (**a**–**c**) Flow cytometry analysis of migLCs, migDC1, migDC2, migDN DCs, resDC1, resDC2, resDN DCs and pDCs in inguinal LNs recovered 24 h or 72 h after FITC application on mouse shaved skin. (**a**) Gating strategy for DC subset identification among single alive cells after exclusion of CD3^+^ T lymphocytes. (**b**) GILZ expression levels in migDC subsets from SDLNs recovered 24 h post FITC application. (**c**) GILZ expression levels in migLCs from SDLNs recovered 72 h post FITC application. (**b**,**c**) Data were normalized relative to FITC^−^ migLCs and expressed as median with interquartile range of normalized delta MFIs. Open symbols represent the values for individual mice. Data are from 2 independent experiments with a total of *n* = 7 mice per group (male mice, C57BL/6J background). Statistical analysis was performed using the non-parametric Mann–Whitney U test to compare GILZ levels in cells from non-treated and FITC-treated flanks. Significance was defined as: *p* < 0.01 **.

We next assessed whether DC exposure to acute skin inflammation translated into alterations of GILZ levels in migDCs that reach SDLNs. LNs were recovered 24 h post FITC application, a time-point optimal for dermal DC migration. DC subsets were identified as previously (Figure 4a) and further divided into inflammation-exposed FITC-positive (FITC^+^) DCs and control FITC-negative (FITC^−^) DCs. No differences were detected regarding MHC II levels between FITC^+^ and FITC^−^ DCs, whatever the subset (Supplemental Figure S3a). GILZ levels were similar between FITC^+^ and FITC^−^ migDC subsets except for migDC1, for which GILZ levels were lower in FITC^+^ that in FITC^−^ cells (Figure 4b and Supplemental Figure S3b). Additionally, migDCs expressed GILZ levels like those detected in resDC2 (Figure 4b and Supplemental Figure S3c), and resDCs and pDCs expressed similar GILZ levels in FITC-treated mice as those observed in untreated mice (data not shown). Thus, this supports the conclusion that only migDC1 display reduced GILZ levels in the context of FITC-induced inflammation. As LCs require more time than dermal DCs to reach the SDLNs [38], we also verified the levels of GILZ in migLCs at a later time point, i.e., 72 h post-FITC application. This analysis confirmed that there were no differences in GILZ levels between FITC^+^ and FITC^−^ migLCs (Figure 4c). Taken together, these results show that skin DC exposure to acute inflammation results in a selective reduction of GILZ levels in migDC1 but not in the other migLC/DC subsets.

### 3.5. GILZ Expression Is Maintained in Skin and SDLN DC Subsets in the Context of Chronic Inflammation

**Figure 5:**
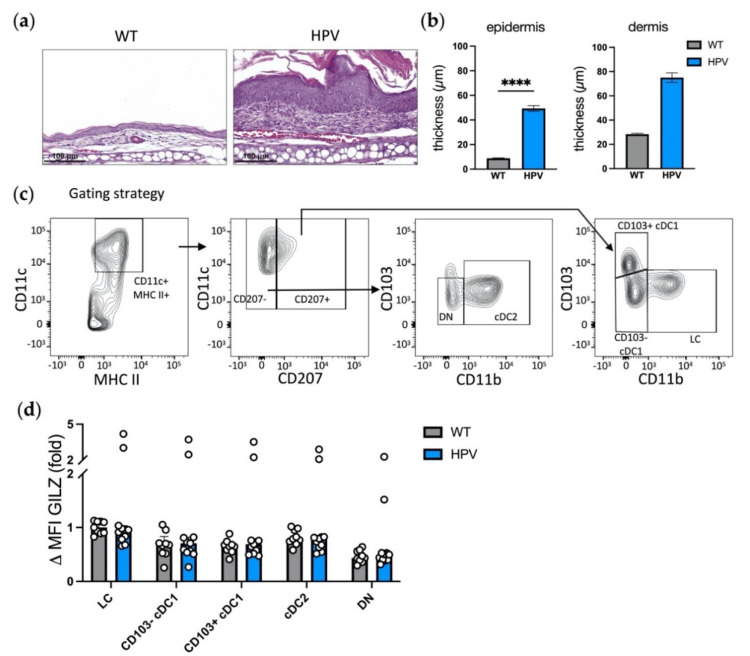
Assessment of GILZ expression levels in skin DC subsets in the context of HPV-associated chronic inflammation. (**a**) Representative images for hematoxylin and eosin coloration of ear skin sections from HPV-transgenic mice (HPV) or their littermate controls (WT). Scale bar = 100 µm. (**b**) Epidermis and dermis ear thickness expressed as mean +/− SEM. At least 10 measures were performed for each sample. Data are from *n*=11 WT and 14 HPV mice for the epidermis and n = 2 WT and *n* = 6 HPV mice for the dermis. (**c**,**d**) Flow cytometry analysis of skin LCs, CD103^−^ cDC1, CD103^+^ cDC1, cDC2 and DN DCs. (**c**) Gating strategy for DC subsets identification among alive single cells after exclusion of CD3^+^ T lymphocytes. (**d**) GILZ expression levels in skin DC subsets. Data were normalized relative to WT LCs and are expressed as median with interquartile range of normalized delta MFIs. Open symbols represent the values for individual mice. Data are from 4 independent experiments with a total *n* = 9 WT mice and *n* = 11 HPV mice (3 male and 6 female WT mice; 3 male and 8 female mice; FVB/N background). Statistical analysis was performed using the non-parametric Mann–Whitney U test to compare GILZ levels in cells from WT and HPV mice. Significance was defined as: *p* < 0.0001 ****.

We next sought to evaluate the impact of chronic inflammation on GILZ expression by skin DCs. We took advantage of a transgenic mouse model of HPV-induced dysplasia that is associated with skin inflammation [33,39]. Skin biopsies were harvested from HPV mice and their littermate controls (WT). Histology assessment confirmed marked skin inflammation, with increased epidermis and dermis thickness (Figure 5a,b). DC subsets were identified (Figure 5c) and their analysis showed that dysplasia-associated inflammation was driving MHC II level diminution in LCs and skin DC subsets (Supplemental Figure S4a), as expected [33,40]. As for GILZ expression, we first confirmed that LCs and cDC2 expressed the highest GILZ levels among skin DCs in the FVB/N background (Figure 5d and Supplemental Figure S4b, DC subsets from WT mice), as in the C57BL/6J background (Figure 1). Further analysis revealed that skin DC subsets displayed similar GILZ levels in HPV mice as in their controls (Figure 5d). Of note, in one experiment, we observed a strong GILZ expression in DC subsets from HPV mice (see outliers in Figure 5b and Experiment 2 in Supplemental Figure S4b), without an identified cause. Thus, HPV-associated chronic inflammation was associated with a maintenance of GILZ expression in LCs and skin DC subsets, as opposed to acute inflammation.

**Figure 6:**
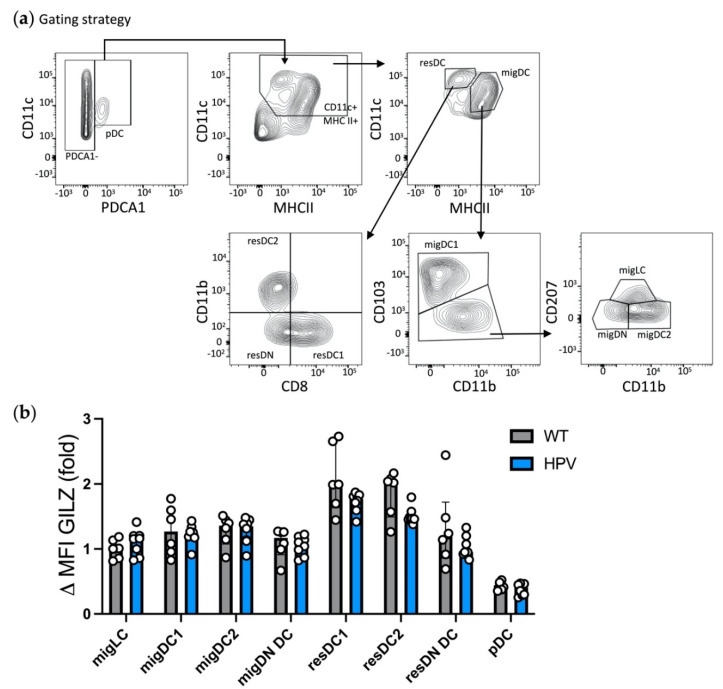
Assessment of GILZ expression levels in LN DC subsets at steady state or in the context of HPV-associated chronic inflammation. (**a**,**b**) Flow cytometry analysis of migLCs, migDC1, migDC2, migDN DCs, resDC1, resDC2, resDN DCs and pDCs. (**a**) Gating strategy for DC subset identification among alive single cells. (**b**) GILZ expression levels in LN DC subsets. Data were normalized relative to WT migLCs and are expressed as median with interquartile range of normalized delta MFIs. Open symbols represent the values for individual mice. Data are from 2 independent experiments with *n* = 6 WT and 8 HPV mice (1 male and 5 female WT mice; 1 male and 7 female HPV mice; FVB/N background). Statistical analysis was performed using the non-parametric Mann–Whitney U test to compare GILZ levels in cells from WT and HPV mice.

We further investigated whether HPV-associated chronic inflammation would alter GILZ expression in DC subsets recovered from SDLNs (Figure 6a). While MHC II expression levels were reduced in SDLN DCs from HPV mice as compared to their controls (Supplemental Figure S5a) as expected from our previous work [33], no alteration of GILZ expression levels were detected, whatever the DC subset analyzed (Figure 6b). Thus, these data demonstrate that the HPV-associated chronic inflammation affects DC subset phenotype without modifying their level of GILZ expression.

### 3.6. GILZ Is Expressed in TIDC Subsets and Its Levels Are Altered in TDLNs

**Figure 7:**
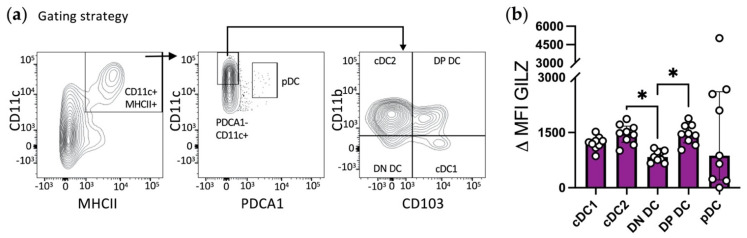
Assessment of GILZ expression levels in TIDC subsets. (**a**,**b**) Flow cytometry analysis of CD11b^−^ CD103^+^ cDC1, CD11b^+^ CD103^−^ cDC2, CD11b^−^ CD103^−^ (DN) DCs, CD11b^+^ CD103^+^ (double positive, DP) cells and pDCs. (**a**) Gating strategy for tumor DC subset identification among CD45^+^ alive single cells. (**b**) GILZ expression level, in tumor DC subsets. Data were normalized relative to cDC1 and are expressed as median with interquartile range of delta MFIs. Data are from 2 independent experiments with a total of *n* = 9 tumor-bearing mice (male mice; C57BL/6J background). Statistical analysis was performed using the Kruskal–Wallis test to compare the different groups. Significance was defined as: *p* < 0.05 *.

Finally, we sought to investigate GILZ expression in DC subsets recovered from solid tumors. TC-1 cells were engrafted subcutaneously to mice, and tumors were recovered after 17 days for TIDC analysis. Five types of DCs were identified, including pDCs, CD11b^+^ CD103^−^ cDC2, CD11b^−^ CD103^+^ cDC1, CD11b^−^ CD103^−^ (DN) DCs (Figure 7a), as previously described [41,42]. We also identified CD11b^+^ CD103^+^ (double positive, DP) cells that we included in our analysis. No CD207^+^ cells were found in the TME (data not shown). Among the TME DC subsets, cDC2, cDC1 and DP cells expressed the highest levels of GILZ while DN DCs displayed the lowest expression (Figure 7b and Supplemental Figure S6). pDCs expressed very heterogenous GILZ levels, from minute to very high. Thus, GILZ expression was detected in the TIDC subsets, the highest protein levels being detected in cDC1 and cDC2, as well as in the DP DCs.

**Figure 8:**
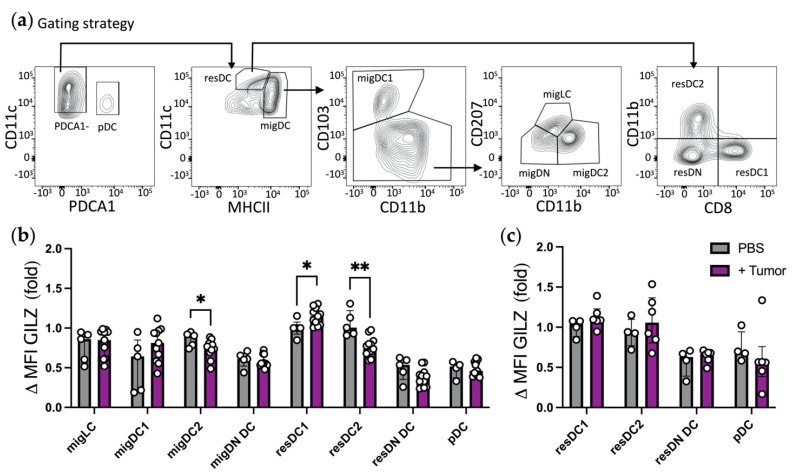
Assessment of GILZ expression levels in DC subsets from TDLNs. (**a**–**c**) Flow cytometry analysis of migLCs, migDC1, migDC2, migDN DCs, resDC1, resDC2, resDN DCs and pDCs of tumor-bearing (tumor) or control (PBS) mice. (**a**) Gating strategy for DC subsets identification among alive single cells. (**b**,**c**) GILZ expression level in LN (**b**) and spleen (**c**) DC subsets. Data were normalized relative to resDC1 from control mice and are expressed as median with interquartile range of normalized delta MFIs. Data are from (**b**) 3 independent experiments with a total of *n* = 5 PBS mice and *n* = 11 tumor-bearing mice or (**c**) 2 independent experiments with a total of *n* = 4 PBS mice and *n* = 6 tumor-bearing mice (male mice; C57BL/6J background). (**b**) For one PBS mouse, there were not enough pDCs for MFI analysis, therefore only 4 symbols are depicted. Statistical analysis was performed using the non-parametric Mann–Whitney U test to compare GILZ levels in cells from PBS mice and tumor-bearing mice. Significance was defined as: *p* < 0.05 * and *p* < 0.01 **.

We next assessed the levels of GILZ expression in DC subsets from TDLNs as compared to inguinal LNs from PBS-injected control mice (Figure 8a). First, we observed similar GILZ levels in migLCs and pDCs recovered from TDLNs and control LNs as expected (Figure 8b and Supplemental Figure S7a). GILZ levels were reduced in migDC2 from TDLNs as compared to control LNs, while migDC1 tended to display higher GILZ levels in TDLNs than in control LNs. As for resDC subsets, resDC1 expressed significantly higher and resDC2 significantly lower GILZ levels in TDLNs than in control LNs. Further assessment of GILZ levels in splenic resDCs revealed no differences between tumor-bearing and control mice, suggesting that the differences observed in TDLNs did not reflect a systemic modulation of GILZ levels in resDC subsets (Figure 8c and Supplemental Figure S7b). Altogether, these results show that GILZ is expressed by TIDCs and that the tumor-context modulates GILZ expression by cDC2 and cDC1 from the TDLNs in an opposite manner.

## 4. Discussion

In this work, we have investigated the expression levels of GILZ in DC subsets from non-lymphoid and lymphoid tissues by multiparametric flow cytometry, at steady state and in different pathologic settings. Our work provides the most exhaustive map of GILZ expression pattern depicted so far, to our knowledge, and unravels the heterogeneity of GILZ expression among these subsets as well as the subset-specificity of its modulation in pathology.

Our results established that GILZ expression levels by DCs vary depending on the subset and tissue of interest at steady state. First, we confirmed our previous observations that resDC1 recovered from lymphoid organs express the highest GILZ levels while pDCs display the lowest ones [25]. By refining our process of DC subsets identification, we also established that resDC2 do not express significantly lower levels of GILZ than resDC1, while resDN DCs do. We further focused on the well-defined DC subsets that survey the skin before migrating to SDLNs [4,43]. In the skin, the highest GILZ levels were found in LCs and dermal cDC2, while CD103^−^/CD103^+^ cDC1 and DN DCs expressed lower GILZ levels. However, the analysis of migDCs recovered in SDLNs revealed similar GILZ expression between the migLC/DC subsets, suggesting that the complex program of maturation undergone by DCs upon migration to SDLNs would include an upregulation of GILZ levels in dermal cDC1 and DN DCs and a downregulation in LCs. Supporting this hypothesis, we have conducted one experiment in which skin and LN DCs were barcoded and further labelled in the same tube. This experiment allowed a direct comparison of GILZ levels expressed in the cells from these tissues and showed that skin cDC2 and migDCs expressed similar levels of GILZ (data not shown). We obtained similar results in the two genetic backgrounds that were explored, namely C57BL/6J and FVB/N, which suggests that they reflect a generalizable process. Taken together, our data show that GILZ expression is not strictly associated with a specific DC subset in the steady state and would rather be conditioned by cell microenvironment.

We have examined the impact of different pathological conditions on GILZ expression levels in DC subsets. We have put a large part of our efforts in the analysis of skin inflammation, as GILZ has been shown to play a substantial role in its regulation in different models in previous studies [44,45,46,47] although no focus was made on DC subsets. First, we have investigated the consequences of an acute inflammation induced upon FITC epicutaneous application [31] on GILZ levels in skin DC subsets. We selected this model because it allows to visually detect the treated area as well as to follow the FITC-exposed DCs and LCs migrating to the SDLNs. Also, we anticipated a broad activation of all the DC subsets upon chemical stress. In this setting, we observed a marked downregulation of GILZ levels in LCs and cDC2 recovered from FITC-exposed skin as compared to those from the contralateral control skin. This reduction could reflect active downregulation of GILZ expression or resistance to GILZ-inducing signals (e.g., endogenous glucocorticoids) in these DC subsets. Such downregulation was however not detected in the FITC^+^ migDC2 nor migLCs recovered from SDLNs as compared to their FITC^−^ counterparts, supporting the conclusion that GILZ downregulation in FITC-exposed LCs and cDC2 was local and transient. We analyzed migLCs 24 h and 72 h post FITC application, which allowed us to detect LCs that were in the epidermis or already on their way to LNs at the time of FITC exposure. While we cannot formally exclude that the FITC^−^ migLCs and migDC2 that we use as internal controls were indirectly exposed to inflammation and thus could have reduced their GILZ expression levels as well, the fact that GILZ levels in FITC^−^ and FITC^+^ migDC2/LCs were similar to those of resDC2, as in the steady state situation, supports our conclusion that GILZ downregulation in LCs and cDC2 is restricted to skin. In terms of functional consequences, as we previously demonstrated that DC macropinocytic activity is modulated by GILZ level [24,25], we propose that such downregulation may favor antigen capture by LCs and dermal cDC2 in the inflamed skin. This is of particular relevance for LCs, that have been reported to contribute to the induction of T cell responses by conveying antigen from the epidermis to SDLNs and transferring it to resDC1 [48]. In addition, as GILZ deficiency was associated with increased production of IL-1, IL-6 and IL-23 by bone-marrow derived DCs (BMDCs) [45], one may anticipate that GILZ downregulation in LCs and dermal cDC2 may support local inflammation by promoting cytokine secretion. Surprisingly, while dermal cDC1 did not show any GILZ downregulation in FITC-treated skin, FITC^+^ migDC1 recovered from SDLNs displayed lower GILZ levels than their FITC^−^ counterparts. As dermal cDC1 express very low levels of GILZ, we propose that this may be explained by a poor upregulation of GILZ in cDC1 migrating from the inflamed skin to SDLNs. Considering previous reports [20,21,24,29], one may anticipate that such GILZ downregulation would favor T-cell priming by migDC1, and thus Th1 and/or Th17 responses, albeit it might also limit antigen cross-presentation [25]. Altogether our results show a subset- and tissue-specific downregulation of GILZ levels in DC subsets in the context of acute skin inflammation. Previous works reported an exacerbation of delayed-type hypersensitivity in GILZ-deficient mice associated with increased Th17 responses [46], suggesting that the maintained expression of GILZ in most DC subsets could contribute to limiting skin inflammation towards chemicals.

We also sought to determine whether GILZ expression would be modulated in DC subsets in the context of chronic skin inflammation. We used transgenic mice that express HPV early genes under the K14 promoter and thus develop HPV-induced dysplasia [32] as a model. These mice display skin inflammation and alteration of DC phenotype that includes a combined downregulation of MHC II expression and upregulation of co-stimulatory molecules [33,39] that is associated with the differentiation of tolerogenic DCs [49]. As opposed to what we were anticipating, we did not detect any increase of GILZ expression in skin and/or SDLN DC subsets, despite skin inflammation and DC phenotype alteration. However, no downregulation of GILZ levels in DCs was detected either, which can be interpreted as the result of an active maintenance of its expression in chronic inflammation. Whether GILZ expression in DC subsets from HPV mice contributes to limiting inflammation would deserve further investigation in mice deficient for [25] or overexpressing GILZ [24] in DCs. In addition, whether GILZ levels are modulated in DCs in the context of other chronic skin inflammations would be worth analyzing. One model of interest would be psoriasis, for which low GILZ levels were reported in patient lesions and associated with the expression of pro-inflammatory cytokines [45]. Interestingly, GILZ has been reported to play either a protective role [45] or a counterintuitive proinflammatory role when overexpressed in all tissues [50] in psoriasis models, supporting the importance of cell-specific regulation of this protein.

We finally have explored GILZ expression in the context of cancer, by analyzing DC subsets in the TME and in TDLNs. Based on our work showing that high levels of GILZ expression in DCs promotes Treg expansion [24], we have chosen the TC-1 tumor model in which Tregs were reported to locally expand in the TME [51]. We first established that the TIDC subsets were expressing GILZ, with similar levels for cDC1 and cDC2. We next assessed the levels of GILZ in DC subsets migrating from the TME to the TDLNs. Our initial plan was to compare the levels of GILZ in DCs migrating from the TME with those in DCs migrating from non-tumoral peripheral tissues, in the same mouse. We have tried to perform such analysis by using TC-1-GFP cells and detect GFP-containing migDCs as previously reported [42]. However, the scarce numbers of cells that were found to contain GFP precluded any conclusive analysis. We thus compared the levels of GILZ in DCs from the TDLNs to those detected in SDLNs of control mice. First, we observed similar GILZ levels in migLCs and pDCs recovered from TDLNs and control SDLNs. As no CD207^+^ LCs were found in the TME and pDCs are not reported to travel from the TME to LNs, we expected no differences in their GILZ levels and used these results as internal controls validating the further comparison of GILZ levels in other DC subsets. Similar GILZ levels being observed in migLCs and pDCs, we enlarged our analysis to the other DC subsets. We were surprised to detect an opposite effect on GILZ expression in the different DC subsets recovered from TDLNs, with a downregulation in mig/resDC2 and an upregulation in resDC1 as compared to their counterparts from control LNs. MigDC1 also tended to display higher GILZ levels in TDLNs than in control LNs. These data suggest an active and DC-subset specific regulation of GILZ levels in the TDLNs. The alteration of GILZ levels in resDCs was unexpected and could have resulted from a distal action of the tumor on DCs in the TDLNs or from a more systemic regulation. We therefore analyzed DC subsets from the spleens of tumor-bearing and control mice. No alteration of GILZ expression being detected in splenic resDCs, this supports the hypothesis that the proximity of the TME imprints TDLNs DCs, an effect that may be mediated by either migDCs or soluble factors. Along this line, tumor supernatants were reported to increase GILZ in BMDCs [30]. However, we did not observe such an upregulation in our experiments (data not shown) and coculture of BMDCs with living tumors did not either [23], suggesting that such an upregulation would depend on the tumor type. Overall, the variation in GILZ levels we observed in the different pathologic conditions were modest, ranging from 10 to 30%, but we have previously established in a model of GILZ overexpression that a 30 to 40% increase in GILZ protein expression drastically modifies DC functions [24,25,26]. In terms of functional consequences, we can hypothesize from previous works that GILZ downregulation in mig/resDC2 would favor Th17 CD4 T-cell priming while its increase in resDC1 would rather promote Treg expansion [20,21,24,29]. Together, this would compromise anti-tumor response efficiency [51,52]. The opposite regulation of GILZ in cDC1 and cDC2 from tumor-bearing mice is of particular interest in view of its contribution in the control of anti-cancer therapy efficiency [23]. Finally, in our experimental model, LCs were not present in the TME. Analyzing the expression and roles of GILZ in LCs from tumor contexts would benefit from genetically engineered mouse models of melanoma or non-melanoma skin cancers [53,54].

Our study provides a general picture in which DC subsets display differential regulation of GILZ protein levels despite being exposed to the same environment. The molecular circuits that control these levels in a subset-specific manner at steady state and in pathology remain elusive, but are likely shaped by distinct proteomes in these subsets [55,56]. GILZ half-life has been reported to be very short [57,58], in support of highly dynamic regulation of its expression. Importantly, GILZ protein levels detected in DC subsets at steady state were not correlated to the *Tsc22d3* gene expression as reported in the ImmGen project [59], supporting the conclusion that post-transcriptional regulation plays a major role in controlling GILZ protein levels in these cells. Along this line, GILZ was previously shown to be subjected to proteasomal degradation in neutrophiles [60], suggesting that such a regulation may also operate in DCs. While prominent contribution of GCs in GILZ tonic expression is expected [61], immunosuppressive cytokines [20,21] or growth factors [29] have been shown to promote GILZ in DCs, suggesting that such factors could be at play in the pathophysiological settings we have investigated. Thus, the high levels of GILZ expressed by LCs in the epidermis could at least partly be explained by keratinocyte expression of TGF-β [62], known to promote GILZ in DCs [20]. TGF-β, that is expressed in TC-1 tumors [63], could also play a role in supporting GILZ expression in tumor DCs. Interestingly, *Tsc22d3* is one of the eight most downregulated genes in microglia from antibiotic-treated mice, supporting the conclusion that microbiota can promote GILZ expression in tissue-resident macrophages [64]. As LCs are in close contact with the skin microbiota, a contribution of commensal bacteria to the control of GILZ levels in this DC-subset could be envisioned. The mechanisms supporting GILZ expression by DCs in the context of chronic inflammation remain elusive. One player in the game could be HGF, known to promote GILZ expression in DCs [29], and whose expression has been reported to increase in chronic inflammation. The receptor for HGF, Met, is expressed in dermal DCs and LCs supporting the hypothesis that HGF-dependent signals could contribute to GILZ expression in chronic inflammation [65]. Also, exposure of monocyte-derived DCs to a cytokine cocktail including IL-6, IL-1β, TNF-α and prostaglandin E2 was reported to promote GILZ expression [66], thus suggesting that combining proinflammatory factors drives GILZ expression as a likely regulatory feedback loop. Regarding GILZ downregulation in acute inflammation and tumor context, it may at least partly result from the activation of the phosphatidylinositol 3-kinase downstream cytokine receptors, as previously reported in other cell subsets [60,67,68]. In the inflamed skin, this could be promoted by the local secretion of cytokines, including IL-1β, TNFα and IL-6 [69,70]. Also, TLR engagement has been reported to reduce GILZ expression in macrophages [71] and LPS exposure decreases GILZ protein levels in DCs [25]. This suggests that the damage signals associated with chemical stress could also contribute to the control of GILZ levels. Identifying the factors that regulate GILZ levels in a DC-specific manner and depending on the pathophysiologic setting will deserve further investigation, that will benefit from conditional GILZ-deficient models.

In conclusion, our study provides an integrated picture of GILZ relative expression in DC subsets, at the protein level. Despite being descriptive, our work conveys important information for further targeted action on GILZ expression. The cartography we have drawn may help deciphering the specific action of GILZ in the different DC subsets and pave the way for its selective targeting in inflammatory contexts and cancers.

## Figures and Tables

**Table 1:** List of mAbs and fixable viability dyes used in the study.

Antigen	Fluorochrome	Supplier	Clone	Dilution
CD3	V500	BD Biosciences	500A2	1/100 or 1/200
CD8α	APC-eFluor 780	eBiosciences	53-6.7	1/100
CD11b	FITC	BD Biosciences	M1/70	1/200
CD11b	PE-CF594	BD Biosciences	M1/70	1/200
CD11b	Super Bright 600	eBiosciences	M1/70	1/200
CD11c	eFluor 450	eBiosciences	N418	1/100
CD11c	PE-Cy7	eBiosciences	N418	1/100
CD16/CD32	Purified	BD Biosciences	2.4G2	1/50
CD45	APC-Cy7	BD Biosciences	30F11	1/200
CD64	PE-Tred	BioLegend	X54-5/731	1/200
CD103	APC-R700	BD Biosciences	M290	1/100
CD207	eFluor 660	eBiosciences	eBioRMUL.2	1/50
PDCA1/CD317	eFluor 450	eBiosciences	eBio927	1/100
MHC II	PerCP Vio700	Miltenyi Biotec	M5/114.15.2	1/200 or 1/500
GILZ	PE	eBiosciences	CFMKG15	1/250
GILZ Isotype	PE	eBiosciences	EBR2A	1/250
Viability dye	eFluor 506	eBiosciences		1/1000

**Table 2:** Markers used to identify skin DC subsets.

LCs	CD103^−^ cDC1	CD103^+^ cDC1	cDC2	DN DC
CD64^−^ CD11c^+^	CD64^−^ CD11c^+^	CD64^−^ CD11c^+^	CD64^−^ CD11c^+^	CD64^−^ CD11c^+^
MHC II^+^ CD11b^hi^ CD207^+^ CD103^int^	MHC II^+^ CD11b^lo^ CD207^+^ CD103^−^	MHC II^+^ CD11b^lo^ CD207^+^ CD103^+^	MHC II^+^ CD11b^+^ CD207^−^ CD103^−^	MHC II^+^ CD11b^−^ CD207^−^ CD103^−^

**Table 3:** Markers used to identify migDC and resDC subsets in SDLNs.

**migLCs**	**migDC1**	**migDC2**	**migDN DCs**	**resDC1**	**resDC2**	**resDN DCs**	**pDCs**
CD11c^+^ MHCII^hi^ CD11b^hi^ CD207^+^ CD103^−^	CD11c^+^ MHCII^hi^ CD11b^lo^ CD207^+^ CD103^+^	CD11c^+^ MHCII^hi^ CD11b^+^ CD207^−^ CD103^−^	CD11c^+^ MHCII^hi^ CD11b^−^ CD207^−^ CD103^−^	CD11c^hi^ MHCII^lo^ CD11b^−^ CD8α^+^	CD11c^hi^ MHCII^lo^ CD11b^+^ CD8α^−^	CD11c^hi^ MHCII^lo^ CD11b^−^ CD8α^−^	CD11c^lo^ CD11b^−^ PDCA1^+^

## Data Availability

All data presented within this study is available within the manuscript.

## Supplementary Materials

The following are available online at https://www.mdpi.com/article/10.3390/cells10113153/s1, Table S1: Antibody panel used for each experiment, Figure S1: GILZ expression levels in LN DC subsets at steady state in individual experiments, Figure S2: Assessment of MHC II and GILZ expression levels in skin DC subsets upon acute inflammation, Figure S3: Assessment of MHCII and GILZ expression levels in LN DC subsets in the context of FITC-induced acute inflammation, Figure S4: Assessment of MHC II and GILZ expression levels in skin DC subsets in the context of HPV-associated chronic inflammation, Figure S5: Assessment of MHC II and GILZ expression levels in LN DC subsets in the context of HPV-associated chronic inflammation, Figure S6: GILZ expression levels in TIDC subsets in individual experiments, Figure S7: Assessment of GILZ expression levels in DC subsets from TDLNs.
