# Supplementary material for: Multi-Tissue Characterization of GILZ Expression in Dendritic Cell Subsets at Steady State and in Inflammatory Contexts
Source: Cells. 2021 Nov 13;10(11):3153. doi: 10.3390/cells10113153 (PMC8623566; doi:10.3390/cells10113153)
Supplement: Supplementary file 1 [file cells-10-03153-s001.zip › cells-1419631-SI.pdf]

cells

MDPI

Supplemental Table S1. Antibody panel used for each experiment.

Antigen	Fluorochrome	Figure 1	Figure 2	Figure 3	Figure 4	Figure 5	Figure 6	Figure 7	Figure 8
Viability dye*	eFluor 506	x	x	x	x	x	x	x	x
CD3*	V500	x	x	x	x	x	x	x	x
CD8α	APC-eFluor 780		x		x		x		x
CD11b	FITC		x			x	x	x	x
CD11b	PE-CF594				x				
CD11b	Super Bright 600	x		x					
CD11c	eFluor 450	x		x					
CD11c	PE-Cy7		x		x	x	x	x	x
CD45	APC-Cy7							x	
CD64	PE-Tred	x		x					
CD103	APC-R700	x	x	x	x	x	x	x	x
CD207	eFluor 660	x	x	x	x	x	x	x	x
PDCA 1	eFluor 450		x		x		x	x	x
MHC II	PerCP Vio700	x	x	x	x	x	x	x	x
GILZ	PE	x	x	x	x	x	x	x	x
FITC painting	FITC			x	x				

* Exclusion of dead cells and CD3 T cells was performed using the same channel.

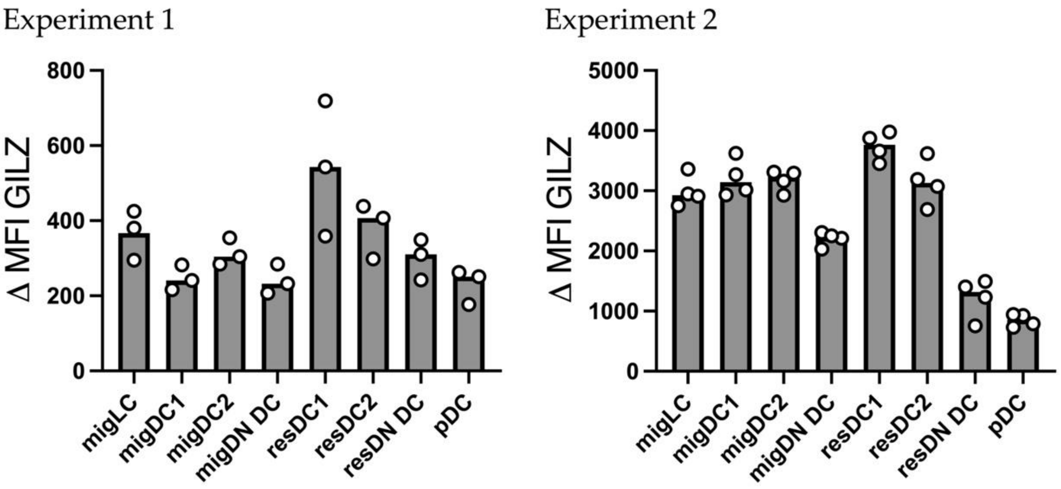

Supplemental Figure S1. GILZ expression levels in LN DC subsets at steady state in individual experiments. GILZ expression levels in LN DC subsets as median of delta MFIs in experiments 1 and 2 used for Figure 2. Open symbols represent the values for individual mice.

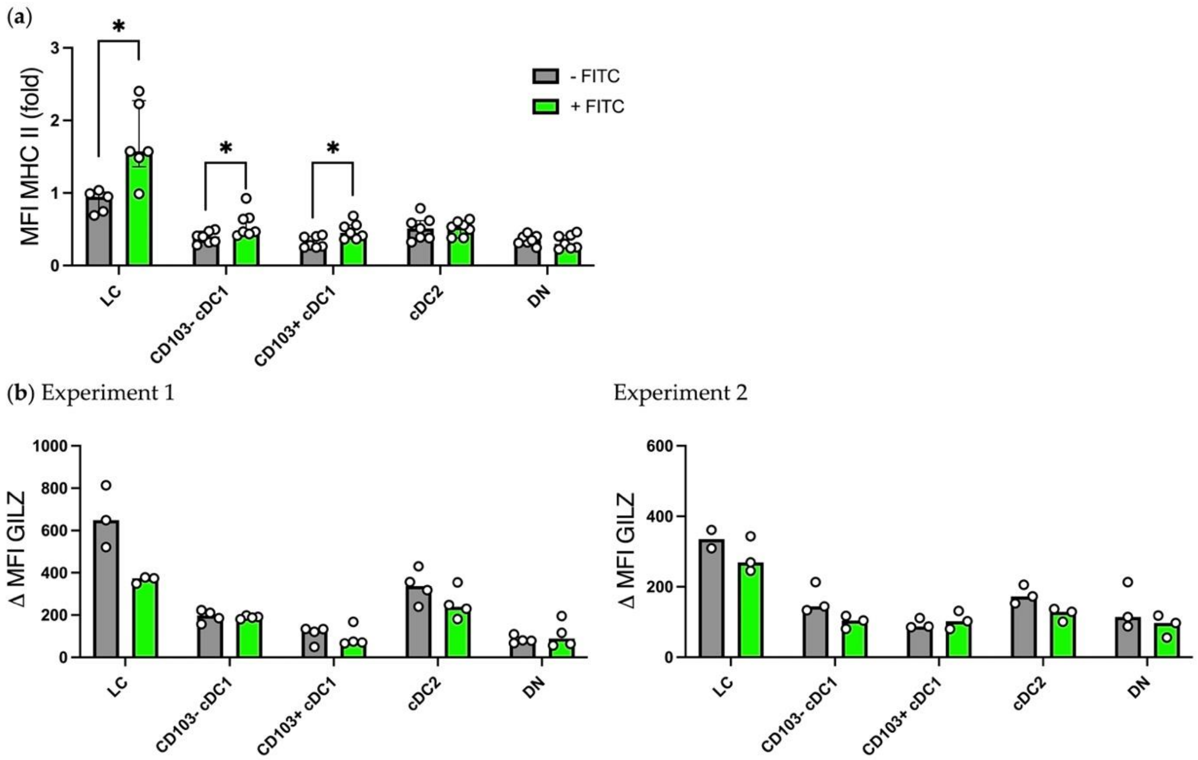

Supplemental Figure S2. Assessment of MHC II and GILZ expression levels in skin DC subsets upon acute inflammation. (**a**) MHC II expression levels in skin DC subsets shown in Figure 3c. Data were normalized relative to FITC- LCs and are expressed as median with interquartile range of normalized MFIs**.** Statistical analysis was performed using the nonparametric Mann-Whitney U test to compare MHC II levels in DC subsets from non-treated and FITC-treated flanks. Significance was defined as: $p < 0.05$ *. (**b**) GILZ expression levels in skin DC subsets as median of delta MFIs in experiments 1 and 2 used for Figure 3c. Open symbols represent the values for individual mice.

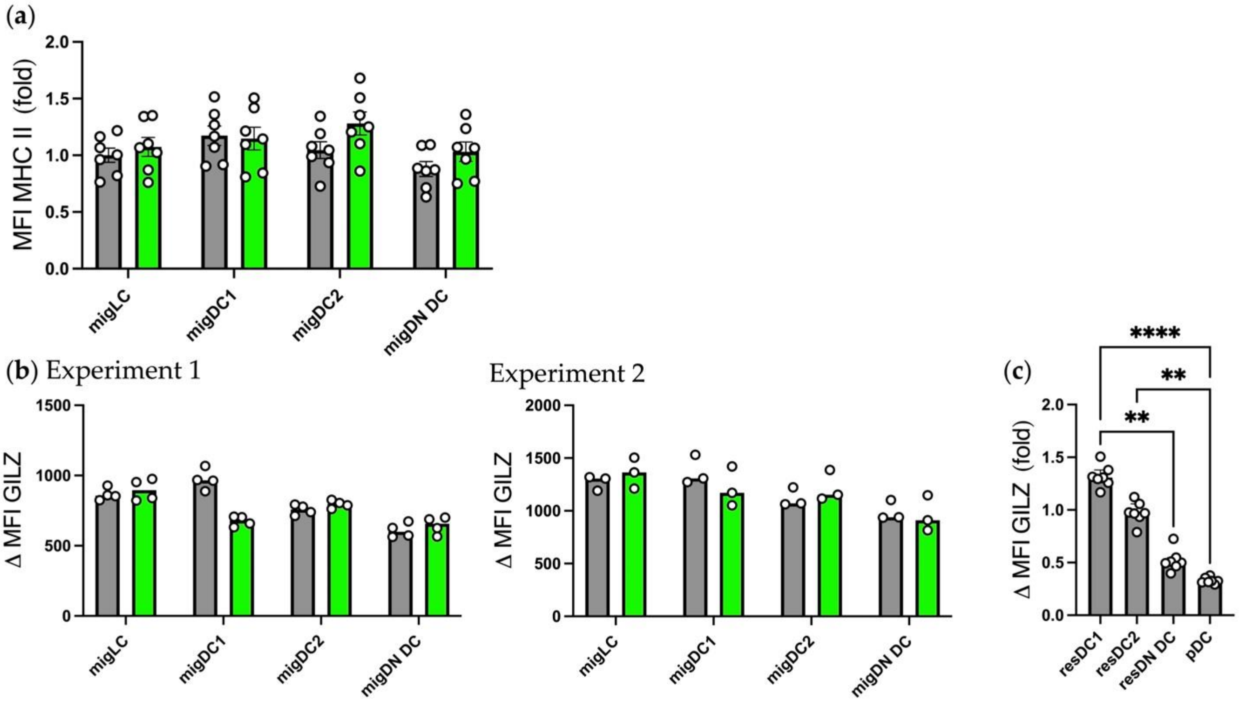

Supplemental Figure S3. Assessment of MHCII and GILZ expression levels in LN DC subsets in the context of FITC-induced acute inflammation. (**a**) MHC II expression levels in LN migDC subsets shown in Figure 4b. Data were normalized relative to FITC$^-$ migLCs and expressed as median with interquartile range of normalized MFIs. (**b**) GILZ expression levels in migDC subsets for experiments 1 and 2 shown in Figure 4b, expressed as median of delta MFIs. (**c**) GILZ relative levels in resDC subsets from SDLNs recovered 24h post FITC application. Data were normalized relative to FITC$^-$ migLCs (see Figure 4b) and expressed as median with interquartile range of normalized delta MFIs. Open symbols represent the values for individual mice. Statistical analysis was performed using the non-parametric Kruskal-Wallis test to compare the different groups. Significance was defined as: $p < 0.01$ ** and $p < 0.0001$ ****.

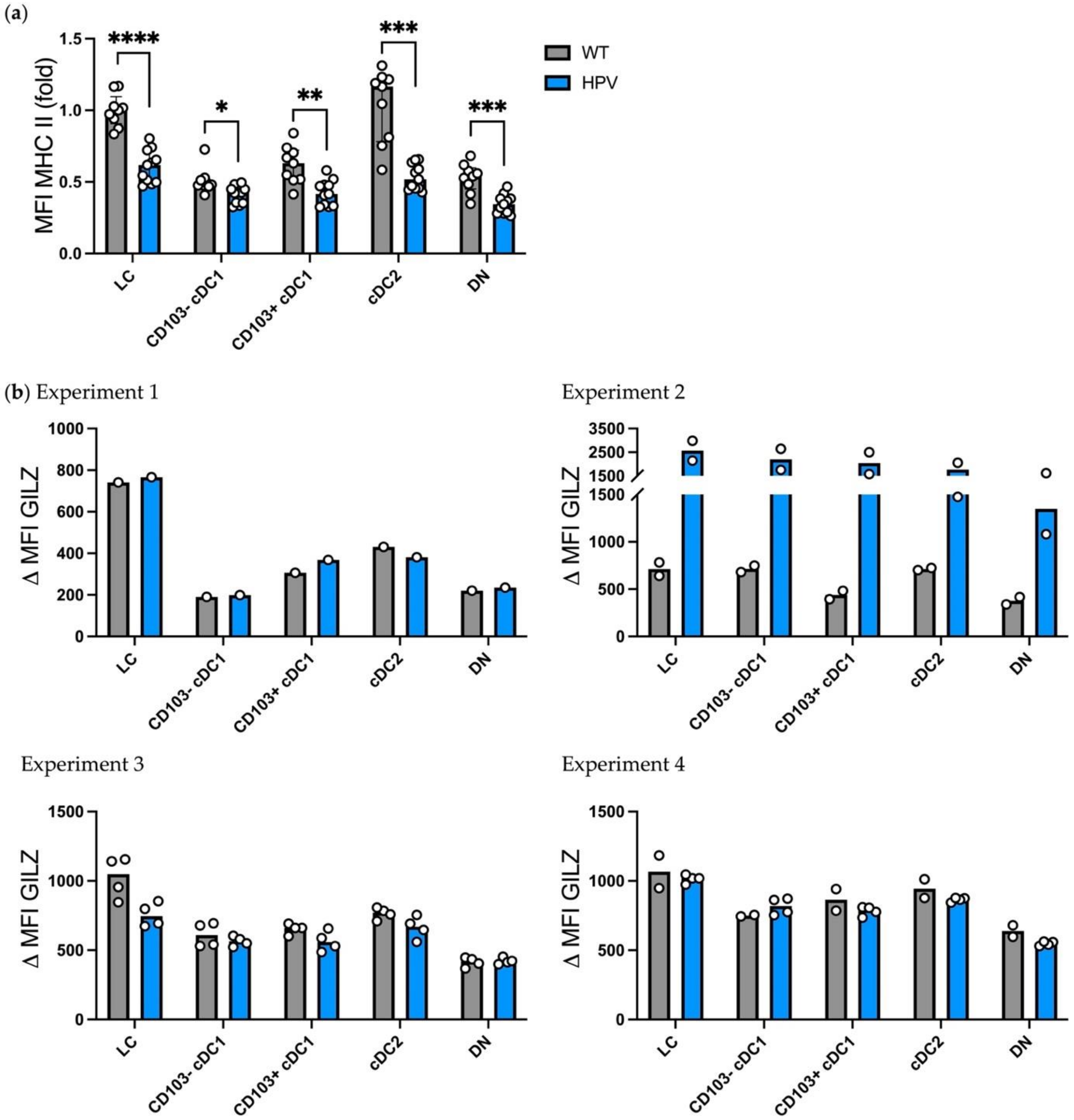

Supplemental Figure S4. Assessment of MHC II and GILZ expression levels in skin DC subsets in the context of HPV-associated chronic inflammation. (**a**) MHC II expression levels in skin DC subsets shown in Figure 5d. Data were normalized relative to WT LCs and are expressed as median with interquartile range of normalized MFIs. Statistical analysis was performed using the nonparametric Mann-Whitney U test to compare MHC II levels in DC subsets from WT and HPV mice. Significance was defined as: $p < 0.05$ *, $p < 0.01$ **, $p < 0.001$ ***, $p < 0.0001$ ****. (**b**) GILZ expression levels in skin DC subsets as median of delta MFIs in the 4 experiments used for Figure 5d. Open symbols represent the values for individual mice.

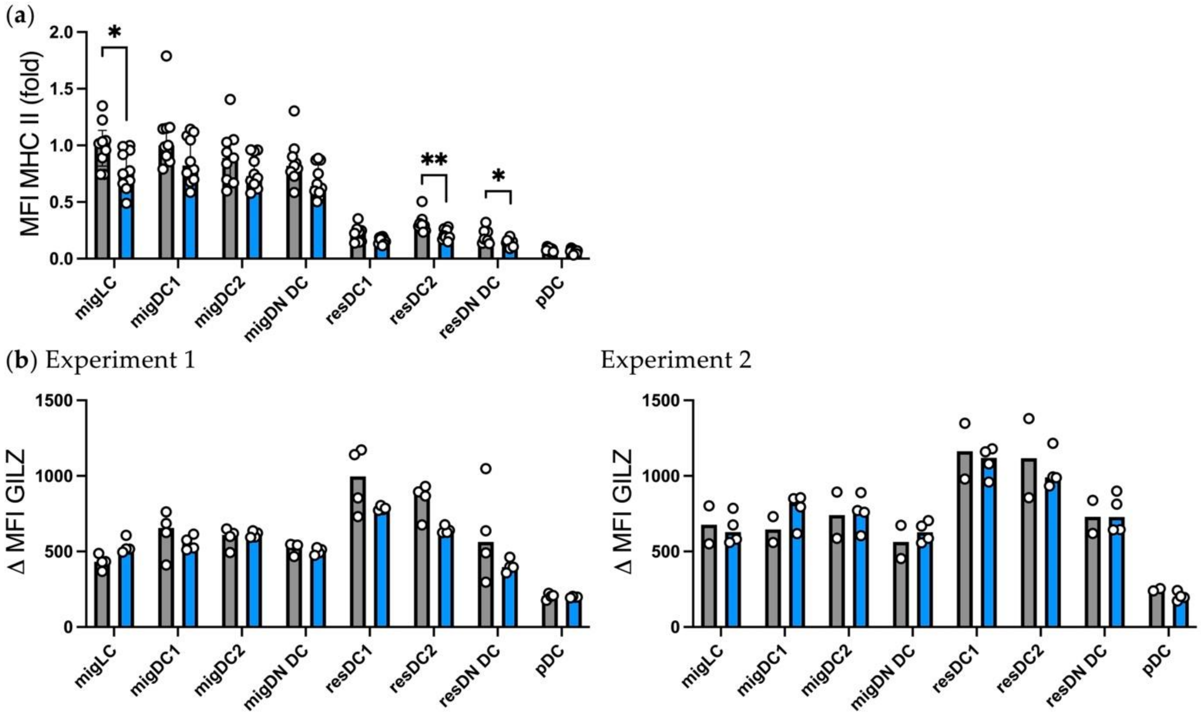

Supplemental Figure S5. Assessment of MHC II and GILZ expression levels in LN DC subsets in the context of HPV-associated chronic inflammation. (**a**) MHC II expression levels in LN DC subsets shown in Figure 6b. Data were normalized relative to WT migLCs and are expressed as median with interquartile range of normalized MFIs**.** Data are from 3 independent experiments with n=9 WT (3 male mice and 6 female mice) and 10 HPV mice (2 male mice and 8 female mice) (FVB/N background). Statistical analysis was performed using the nonparametric Mann-Whitney U test to compare MHC II levels in LN DC subsets from WT and HPV mice. Significance was defined as: $p < 0.05$ * and $p < 0.01$ **. (**b**) GILZ expression levels in LN DC subsets as median of delta MFIs in the 2 experiments used for Figure 6b. Open symbols represent the values for individual mice.

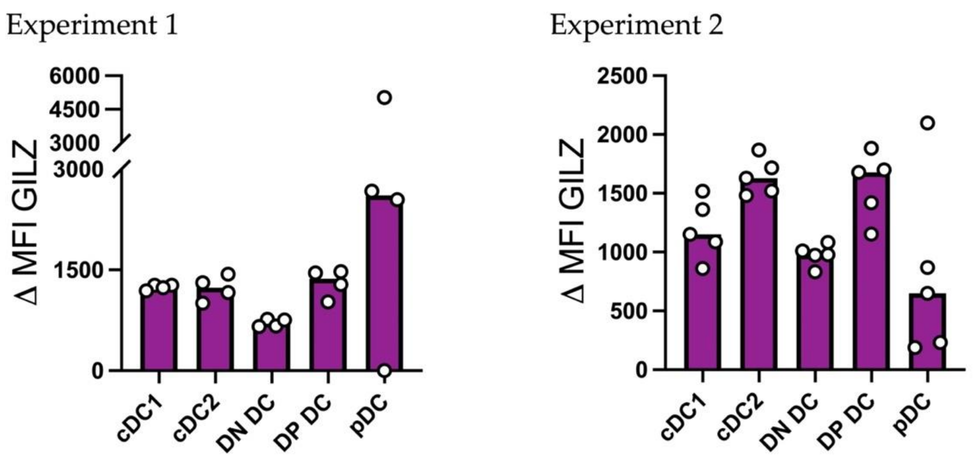

Supplemental Figure S6. GILZ expression levels in TIDC subsets in individual experiments. GILZ expression levels in TIDC subsets as median of delta MFIs in experiments 1 and 2 used for Figure 7. Open symbols represent the values for individual mice.

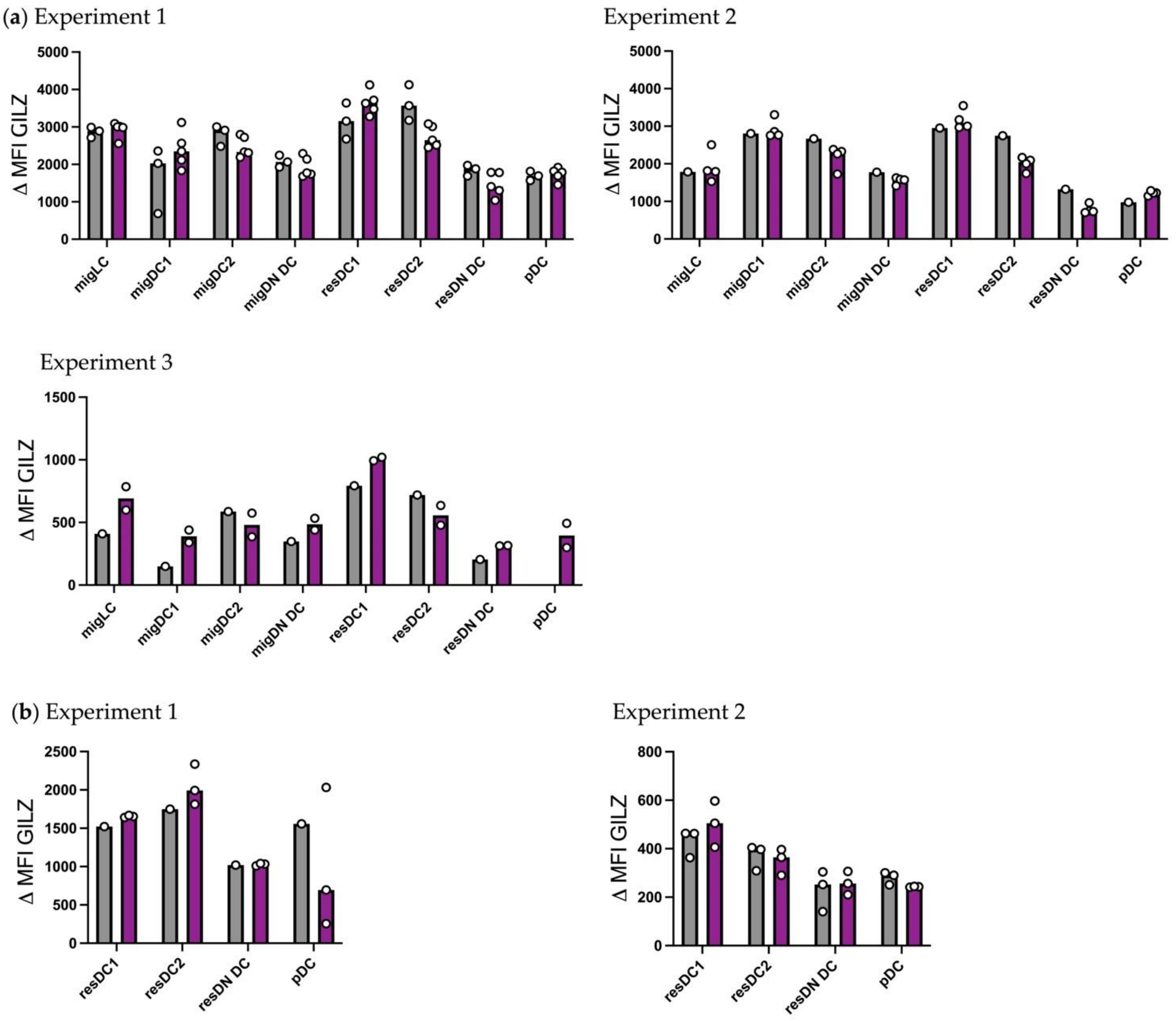

Supplemental Figure S7. Assessment of GILZ expression levels in DC subsets from TDLNs. (**a, b**) GILZ expression levels in LN (**a**) and spleen (**b**) DC subsets as median of delta MFIs in the independent experiments used for Figure 8b and c. Open symbols represent the values for individual mice. In the third experiment used for Figure 8b (**a**), there were not enough pDCs for MFI analysis in the WT LN, therefore no symbol is depicted for WT pDCs in this experiment.
